# "Coercion Experience Scale" (CES) - validation of a questionnaire on coercive measures

**DOI:** 10.1186/1471-244X-10-5

**Published:** 2010-01-14

**Authors:** Jan Bergk, Erich Flammer, Tilman Steinert

**Affiliations:** 1 Center for Psychiatry Suedwuerttemberg, Ulm University, Ravensburg-Weissenau, Germany

## Abstract

**Background:**

Although the authors of a Cochrane Review on seclusion and mechanical restraint concluded that "there is a surprising and shocking lack of published trials" on coercive interventions in psychiatry, there are only few instruments that can be applied in trials. Furthermore, as main outcome variable safety, psychopathological symptoms, and duration of an intervention cannot meet the demand to indicate subjective suffering and impact relevant to posttraumatic stress syndromes. An instrument used in controlled trials should assess the patients' subjective experiences, needs to be applicable to more than one intervention in order to compare different coercive measures and has to account for the specific psychiatric context.

**Methods:**

The primary version of the questionnaire comprised 44 items, nine items on restrictions to human rights, developed on a clinical basis, and 35 items on stressors, derived from patients' comments during the pilot phase of the study. An exploratory factor analysis (EFA) using principal axis factoring (PAF) was carried out. The resulting factors were orthogonally rotated via VARIMAX procedure. Items with factor loadings less than .50 were eliminated. The reliability of the subscales was assessed by calculating Cronbach.

**Results:**

Data of 102 patients was analysed. The analysis yielded six factors which were entitled "Humiliation", "Physical adverse effects", "Separation", "Negative environment", "Fear" and "Coercion". These six factors explained 54.5% of the total variance. Cronbach alpha ranged from .67 to .93, which can be interpreted as a high internal consistency. Convergent and discriminant validity yielded both highly significant results (r = .79, p < .001, resp. r = .38, p < .001).

**Conclusions:**

The "Coercion Experience Scale" is an instrument to measure the psychological impact during psychiatric coercive interventions. Its psychometric properties showed satisfying reliability and validity. For purposes of research it can be used to compare different coercive interventions. In clinical practice it can be used as a screening instrument for patients who need support after coercive interventions to prevent consequences from traumatic experiences. Further research is needed to identify possible diagnostic, therapeutic or prognostic implications of the total score and the different subscales.

**Trial registration:**

Current Controlled Trials ISRCTN70589121

## Background

During psychiatric in-patient treatment coercive measures such as seclusion, mechanical or physical restraint, or net beds are considered as interventions of last resort [1]. Their use needs to be carefully reviewed and monitored, representing the greatest restriction on a person's freedom in psychiatry [2]. Rates of admissions exposed to seclusion and mechanical restraint vary widely with rates ranging from 0% to 66% [3-9]. The variations in the use of seclusion or mechanical restraint point to powerful local effects [3,10-12]. Restrictiveness of psychiatric containment methods are embedded in wider national cultures, rather than an isolated tradition of professional psychiatric practice [13]. Within the scope of emphasis on evidence based medicine in psychiatry it seems doubtful that local traditions instead of scientific evidence determine the kind of intervention [14]. Lately, prominent international recommendations have aimed to restrict the use of seclusion and restraint, and reminded clinicians that these measures should only be used in exceptional cases [8]. The "least restrictive alternative" is recommended [1,15-21].

A review published in 2006 searched the literature from 1985 to 2002 and yielded that only insufficient evidence is available to determine whether seclusion and restraint are safe and/or effective interventions for the short-term management of disturbed/violent behaviour in adult psychiatric inpatient settings. These interventions should therefore be used with caution and only as a last resort once other methods of calming a situation and/or service user have failed [20].

However, evidence about what kind of intervention is least restrictive is only scarcely available. A Cochrane Review on "seclusion and restraint for people with serious mental illnesses" concluded that "no controlled studies exist that evaluate the value of seclusion and restraint in those with serious mental illness. (...) Continuing use of seclusion or restraint must therefore be questioned from within well-designed and reported randomised trials that are generalisable to routine practice" [7]. Compared to how restrictive psychiatric coercive interventions are, "there is a surprising and shocking lack of published trials assessing the effects of secluding and restraining people with schizophrenia or similar psychotic illnesses" [7]. On the other hand, ethical and methodological difficulties of randomised controlled trials (RCT) on coercive interventions have only recently been addressed [22]. Safety, psychopathological symptoms, and duration of an intervention as main outcome variables cannot meet the demand to indicate subjective suffering and impact relevant to posttraumatic stress syndromes [22]. Furthermore, patients' complaints about coercive interventions as well as most public debates do not primarily address objective characteristics such as safety, efficacy, or duration of these measures but focus on subjective feelings of humiliation, punishment, and traumatisation [23-28]. Therefore from the service user's perspective coercive measures do not primarily represent a problem of safety but a problem of human rights and of the subjective experience of strain [17,29].

Up to now, instruments developed to measure this subjective impact focus on posttraumatic stress disorder of survivors of war, victims of torture, or political detainees [30,31]. Instruments measuring human rights status in general do not account for specific psychiatric settings [32-34]. Another instrument refers to only one intervention [35].

With the MacArthur Admission Experience Interview and Survey (AEI and AES), an instrument was introduced which is designed to assess the patients' subjective experience of coercion, but only referring to the hospital admission process and is therefore not suitable to measure the impact of coercive interventions. This instrument has increasingly been used in the last years [36-38]. Similarly, in order to yield more evidence on different kinds of coercive interventions, an instrument is needed to assess patients' subjective experience during coercive interventions like seclusion or mechanical restraint. Validated scales that assess and compare freedom-restricting coercive measures such as seclusion and restraint are not available yet. An instrument for the assessment of coercion during coercive interventions in psychiatry has to:

1. assess the patients' subjective experiences,

2. be applicable to more than one intervention in order to detect differences between two or more coercive interventions,

3. reflect the ethical considerations referring to the restriction of human rights,

4. cover a wide range of interindividual highly varying stressors,

5. account for the specific psychiatric context,

6. constitute a cut-off value that indicates the risk of traumatisation and the critical amount of strain,

7. be concordant with other instruments in self- and staff-member-assessment as external points of reference.

This article describes the development of an assessment tool meeting these demands.

## Methods

The factor analysis reported in this manuscript is a post-hoc analysis of data that was collected within a trial on seclusion and mechanical restraint. The study was approved by the ethical committee of the University of Ulm.

### Definitions

*Seclusion *is defined as an involuntary confinement of a person in a room or an area where the person is physically prevented from leaving [39].

*Mechanical restraint *refers to the use of belts, handcuffs and the like, which restricts the patient's movement or totally prevents the patient from moving [8].

The *index-intervention *was the first coercive intervention after admission. Although there might have been other coercive interventions during the course, the interview focused on the index-intervention.

### Sample/Subjects

We used a probabilistic sampling strategy and screened 233 patients with the primary ICD-10 diagnoses F2, F3 and F6 (schizophrenic disorder, affective disorder, personality disorder) who were admitted to three general psychiatric admission wards between March 2003 and March 2005, and had experienced a coercive intervention. We had to exclude 125 patients. 93 patients did not meet inclusion criteria (Diagnosis n = 22, readmission n = 22, discharge before interview n = 13, voluntary (coercive) measure n = 12, language barrier n = 11, continuation of severe symptoms n = 9, measure not recalled n = 4). 32 patients refused to participate.

It was intended to include 200 patients. In March 2005 the regulating authority of the hospital, the Ministry of Social Affairs of our county, send a directive referring to a visit by the European Committee for the Prevention of Torture and Inhuman or Degrading Treatment or Punishment (CPT). This directive prohibited mechanical restraint without constant observation by staff members. This directive was appreciated by our hospital and we had to change our guideline. For this reason study conditions had changed because before we allowed several exceptions (e.g. no observation while patient sleeps, observation only in 3/4 of an hour per hour of the intervention). Consequently, we had to stop the trial.

The problem of informed consent and competence is discussed in detail in a former publication [22]. The scale was developed in German. The items have been translated into English. Retranslation yielded a high congruence.

### Theoretical considerations and development of the questionnaire

To assess the restrictiveness of coercive interventions we considered two aspects as most relevant: firstly, the restrictions of human rights during coercive interventions, and secondly, the stressors resulting from the coercive intervention. Thus the questionnaire consisted of these two parts. The items referring to restrictions of human rights (HR) and the items referring to the stressors (S) were constructed from different sources.

#### a) Restrictions of human rights

The items on restrictions of human rights were developed on a clinical basis after a literature search. Violation of human rights by coercive interventions and appropriate questions aiming at these specific aspects were discussed during several meetings of a research group. Human dignity, autonomy, freedom of movement, physical inviolability, and limitations of contact with staff and fellow patients were considered to be the most important human rights restricted during coercive interventions. Each aspect of violation of human rights was assessed to what degree it was restricted (a little/moderate/severe/very severe/extreme) and how this was experienced (acceptable/uncomfortable/unpleasant/very unpleasant/extremely unpleasant). The extent was considered to reflect objective conditions, the experience the subjective impact. Human dignity was the only human right that was assessed solely to the extent, because the emotional impact of human dignity was considered to be covered by the extent and therefore has not to be questioned by the experience. Physical inviolability was questioned by the extent of coercion applied during the intervention and how this was experienced.

#### b) Stressors

The part of the questionnaire that focuses on the stressors imposed by coercive interventions was developed by interviews with service users. During a pilot phase of the study, ten patients were interviewed about their experienced stressors and from which they suffered most, after they were exposed to either seclusion or mechanical restraint. The answers yielded 35 stressors. In the main study, these stressors were assessed on a Likert-Scale (not stressful/mildly/moderate/severely/extreme).

### Construction of the first version of the questionnaire

Thus the primary version of the questionnaire comprised 44 items, nine items on restrictions to human rights (HR1-HR9), developed on a clinical basis, and 35 items on stressors (S1-S35), derived from patients' comments during the pilot phase of the study. The questions address restrictions during coercive interventions in detail and were mostly well understood by the patients. Only 41 items entered the factor analysis. The items stressor 6 (pain by belts), stressor 12 (inability to scratch while skin bites), and stressor 27 (I was not able to act freely) were excluded, because stressor 6 and stressor 12 referred only to mechanical restraint and stressor 27 was often misapprehended in the German version.

### Additionally applied instruments

In order to measure the validity of the "Coercion Experience Scale" (CES) following self-assessment instruments within the scope of coercive interventions were applied (for the most part four weeks after the index-intervention, if not mentioned otherwise):

- Visual-analogue-scale (VAS) measuring the global burden of the coercive measure,

- Screening instrument for Posttraumatic Stress Disorder (PTSD) [40],

- Patient satisfaction [41] and

- Impact of Event-Scale (IES-R) [42,43]. The IES-R was applied one year after the index-intervention.

Similar to the above mentioned VAS of the patients' assessment, staff members assessed a

- Visual-analogue-scale measuring the assumed patient's global burden during the index-intervention.

Additionally, the duration between intervention and interview was assessed. Psychopathological symptoms were measured by selected items of the Positive and Negative Syndrome Scale (PANSS) aggregated in an aggression score with a range from 7 to 28. A high score is indicating a higher level of aggression.

### Exploratory factor analysis and item analyses

The construction of the questionnaire was carried out in two steps. In order to uncover the underlying structure of the items, an exploratory factor analysis (EFA) using principal axis factoring (PAF) was carried out. The factorisability of the correlation matrix for EFA was judged by Kaiser-Meyer-Olkin criterion on the basis of the measure of sampling adequacy. A Bartlett test of sphericity was applied to test whether correlations differ significantly from zero. The number of relevant factors was determined by application of the Kaiser criterion. After extraction, the resulting factors were orthogonally rotated via VARIMAX procedure. From the resulting factor solution, items with factor loadings less than .50 were eliminated. Subsequently, separate item analyses for each factor consisting of the retained items were carried out. The reliability of these subscales was assessed by calculating Cronbach α and those items that decreased α were eliminated.

### Construct validity

Construct validity was evaluated by calculating correlation coefficients between the subscales and a VAS scale measuring strain, a PTSD screening [40], patient satisfaction [41] and the IES-R [42,43]. These scales were all available as self-assessment instruments. Additionally, staff members assessed the global strain during the index-intervention on the same VAS scale like the patients. The evaluation by the staff members served as external reference point.

### Risk of traumatisation

To estimate the risk of traumatisation induced by the coercive intervention, a regression of the total score on the PTSD screening score was carried out. The PTSD screening seemed to be the most appropriate instrument to estimate approximately a cut-off value. The critical value on the CES total scale was calculated by inserting the cut-off point of the PTSD screening in the resulting regression equation.

### Software

StatSoft, Inc. (2007). STATISTICA for Windows (Software-System for data analyses) version 8.0.http://www.statsoft.com.

## Results

102 patients participated. Table 1 shows the sample characteristics. Descriptive statistics showed that patients in the seclusion group had more hospitalisations as an indicator for chronicity, lived apart more frequently and had a higher percentage of pensioners. Patients in the mechanical restraint group were more often married and more often part time employees. Apart from these variables, the sociodemographic data and the psychiatric baseline data did not differ substantially between the intervention groups. Psychopathological symptoms at the time point of the interview were reduced to a large extend. Only in 9 patients "continuation of severe symptoms" as an exclusion criterion had to be used. In total the data is representative for the usual population on admission wards in our hospital.

**Table 1:** Sample characteristics

Patients	Secluded (N = 60)	Mechanically restrained (n = 42)
**Age**		
Mean	40.3	39.3
StdDev.	11.6	12.8

**Gender**		

Male	27 (45.0%)	23 (54.8%)
Female	33 (55.0%)	19 (45.2%)

**Diagnoses (only first diagnosis)**		

F2 (total)	40 (66.6%)	26 (61.9%)
F3	10 (16.7%)	9 (21.4%)
F6	10 (16.7%)	7 (16.7%)

F2 in detail:		
F20	26 (43.3%)	14 (33.3%)
F23	2 (3.3%)	4 (9.6%)
F25	12 (20.0%)	8 (19.0%)

**Chronicity (Number of former hospitalisations)**		

median	5.00	3.00
Min.	0.00	0.00
Max	90	33
Mean	9.85	5.9
StdDev.	17.0	8.2

**Family Status**		

Unmarried	33 (55.0%)	21 (50.0)
Married	6 (10.0%)	11 (26.2%)
Long-term relationship	3 (5.0%)	2 (4.8%)
Divorced	11 (18.3%)	7 (16.7%)
Live apart	6 (10.0%)	1 (2.4%)
widowed	1 (1.7%)	0

**Educational Status**		

No graduation	3 (5.0%)	2 (4.8%)
Secondary	38 (63.3%)	24 (57.1%)
Higher	16 (26.7%)	13 (31.0%)
University degree	3 (5.0%)	3 (7.1%)

**Employment**		

Full-time employee	7 (10.9%)	4 (8.0%)
Part time employee	4 (6.3%)	7 (14.0%)
Unemployed	12 (18.8%)	8 (16.0%)
Registered as jobless	5 (7.8%)	5 (10.0%)
Sheltered workshop	7 (10.9%)	6 (12.0%)
Pensioner	17 (26.6%)	8 (16.0%)
Others	12 (18.8%)	12 (24.0%)

**Psychopathological symptoms**		

Start of intervention (mean)	19.5	22
End of intervention (mean)	9.3	6.9

**Time intervention-interview**		

median	30	27
Min.	3	2
Max	114	404
Mean	33.5	40.1
StdDev.	25.2	62.2

Former coercive interventions		

Former mechanical restraint	46.7%	45.2%
Former seclusion	51.7%	31.0%
Former forced medication	45.0%	42.9%

### Exploratory factor analysis

**Figure 1:**
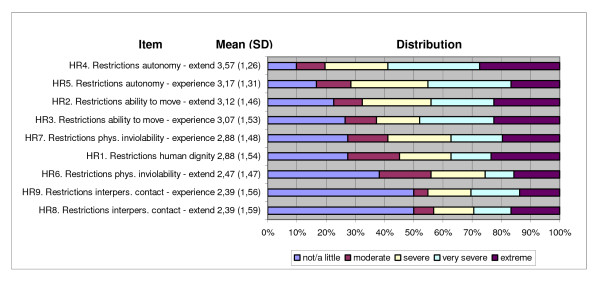
**Characteristics of items I - restrictions of human rights**. Single restrictions sorted by mean.

**Figure 2:**
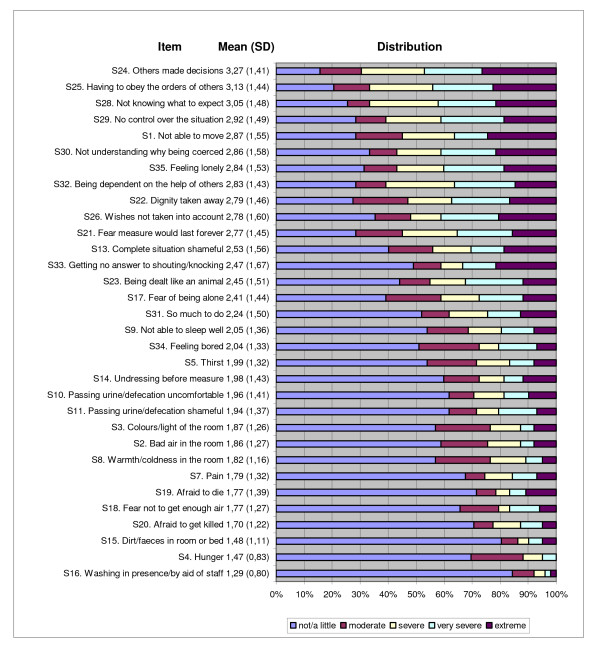
**Characteristics of items II - stressors**. Single stressors sorted by mean.

For N = 102 patients the item characteristics and the parameters of the distributions sorted by mean are shown in figure 1 and 2 (highest score is 5 (extreme stressing/restrictive), lowest score is 1 (not/a little stressing/restrictive)). In a first step, the factorisability of the correlation matrix was checked. The measure of sampling adequacy was .817 and a Bartlett test of sphericity became significant (χ^2 ^= 2786.77, df = 820, p < .001).

### Factor extraction

An explanatory factor analysis (EFA) was carried out using principal axis factoring (PAF). To determine the number of relevant factors, a scree plot was used and the Kaiser criterion (eigenvalue λ ≥ 1.00) was applied. The analysis yielded six factors with λ ≥ 1.00. These six factors explained 54.5% of the total variance of intercorrelations. (Factor 1 "Humiliation": 33.1%, Factor 2 "Physical adverse effects": 7.4%, Factor 3 "Separation": 4.7%, Factor 4 "Negative environment": 3.8%, Factor 5 "Fear": 3.0%, Factor 6 "Coercion": 2.5%. In a subsequent step, the factors were orthogonally rotated via VARIMAX procedure.

### Item elimination

After factorisation, items with factor loadings <.50 were eliminated (S4. Hunger, S5. Thirst, S14. Undressing before beginning of the measure, S15. Dirt/faeces in room/bed, S17. Fear of being alone, S29. No control over the situation, S31. So much to do, S32. Being dependent on the help of others, S33. Getting no answer to shouting and knocking, S34. Feeling bored, S35. Feeling lonely). After elimination of these items with low loadings the structure was assessed during a research group meeting. The factor solution was interpreted and considered as helpful to differentiate different aspects of restrictions during coercive interventions. Table 2 depicts the retained items and their factor loadings.

**Table 2:** Factor solution with items

Factors and items	Factor loading					
**Humiliation**	**Factor 1**	**Factor 2**	**Factor 3**	**Factor 4**	**Factor 5**	**Factor 6**

S24. Others made decisions	.87	-.02	.06	.16	.02	-.02
S25. Having to obey the orders of others	.85	-.01	.02	.10	.08	.15
S22. Dignity taken away	.81	.12	.05	.24	.24	.11
HR3. Restrictions ability to move - experience	.69	.29	.37	-.03	-.02	.00
S26. Wishes not taken into account	.68	.03	.23	.20	.12	.18
S23. Being dealt like an animal	.68	.13	.08	.35	.25	.17
HR1. Restrictions to human dignity	.66	.18	.38	.02	-.08	.21
S3. Not understanding why being exposed to coercion	.65	.19	.16	-.02	.10	.17
HR5. Restrictions to autonomy - experience	.64	.06	.37	-.05	-.03	.09
S28. Not knowing what to expect	.63	.31	.38	.10	.10	.08
HR2. Restrictions to ability to move - extent	.62	.28	.32	-.18	-.03	.17
HR4. Restrictions to autonomy - extent	.60	-.02	.31	.01	-.08	.10
S1. Not able to move	.60	.35	.36	-.01	.05	-.02
S13. Complete situation shameful	.53	.12	.01	.23	.06	.26
S21. Fear measure would last forever	.51	.09	.43	.26	.32	-.21

**Physical adverse effects**						

S1. Passing urine/defecation uncomfortable	.19	.78	.21	.21	.02	.18
S7. Pain	.01	.60	.01	.10	.15	.00
S16. Washing/body hygiene in presence/by aid of staff .28	.28	.51	-.11	.12	.33	-.16
S11. Passing urine/defecation shameful	.30	.50	-.02	.28	-.22	.15

**Interpersonal separation**						

HR9. Restrictions to interpersonal contact - experience .28	.28	.06	.78	.06	.15	.19
HR8. Restrictions to interpersonal contact - extent .32	.32	.05	.68	.14	.15	.23

**Negative environmental influences**						

S8. Warmth/coldness in the room	.09	.25	.16	.65	.05	.06
S3. Colours/light of the room	.32	.26	.06	.60	-.06	-.11
S2. Bad air in the room	.20	.17	-.03	.58	.12	.06
S9. Not able to sleep well	.24	.17	.25	.53	-.17	-.01
S18. Fear not to get enough air	.09	.05	.19	.51	.41	-.05

**Fear**						

S19. Afraid to die	.13	.01	.13	.01	.13	.01
S2. Afraid to get killed	.25	.04	.25	.04	.25	.04

**Coercion**						

HR6. Restrictions to physical inviolability - extent .38	.38	.14	.22	.04	.00	.69
HR7. Restrictions to physical inviolability - experience .41	.41	.11	.24	-.04	.02	.61

### Item analysis

After elimination of those items with low factor loadings, separate item analyses for each factor were carried out. The analysis yielded the following results shown in table 3. Elimination of item S13 (Complete situation shameful) increased Cronbach α for factor 1 (.928 to .930). Item S16 (Washing/Body hygiene only in presence by aid of staff) showed low item difficulty (.08). But as its elimination would decrease reliability (Cronbach α from .724 to .711), item S16 was retained. Standardised α differed maximum .01 to Cronbach α.

**Table 3:** Descriptive statistics for the subscales and the total scale

Factor	Subscale	N	Mean	SD	Median	**Min**.	**Max**.	Cronbach α	Mean inter-item correlation
1	Humiliation	102	36.19	13.15	38.00	12.00	60.00	.93	.53

2	Phys. adv. effects	102	7.12	3.76	6.00	4.00	19.00	.72	.41

3	Separation	102	4.78	3.02	4.00	2.00	10.00	.92	.85

4	Neg. environment	102	9.39	4.63	8.00	5.00	24.00	.78	.43

5	Fear	102	3.49	2.27	2.00	2.00	10.00	.67	.51

6	Coercion	102	5.33	2.73	4.00	2.00	10.00	.83	.72

					64.00	30.00	115.00		

### Descriptive statistics for the subscales

The descriptive statistics for the subscales and the total scale after elimination of those items decreasing Cronbach α are shown in table 3. The scores on subscales 2, 3, 4, 5, 6 are not normally distributed (Lilliefors-p < .01). For subscale 1 the test for departure from normal distribution shows a tendency towards significance (Lilliefors-p < .10). The scores on the total scale can be assumed to be normally distributed (Lilliefors-p > .20).

### Intercorrelations of the subscales

The subscales showed the intercorrelations displayed in table 4.

**Table 4:** Intercorrelations of the subscales (N = 100)

	Humiliation	Physical adverse effects	Separation	Negative environment	Fear	Coercion	Total scale
Humiliation	1.00	0.43***	0.56***	0.41***	0.36***	0.63***	0.93**

Phys. adv. Effects		1.00	0.23*	0.46***	0.23*	0.3**	0.6***

Separation			1.00	0.26**	0.34***	0.84**	0.69***

Neg. environment				1.00	0.22*	0.27**	0.61***

Fear					1.00	0.36***	0.49***

Coercion						1.00	0.74***

Total scale							1.00

*** p < .001 ** p < .01 * p < .05

### Construct validity

To assess the construct validity, correlation coefficients between the subscales, the total scale, a VAS scale measuring strain, a PTSD screening, the Rosenberg-self-esteem-scale, patient satisfaction and the IES-R were calculated. As external point of reference, the correlation coefficient between the total scale and the VAS assessing the global strain during the index-intervention as perceived by staff-members was determined. The correlations are depicted in table 5. The subscales showed, as expected, positive correlations for VAS Global Strain (self-assessment), PTSD-screening and negative correlations for patient satisfaction. But contrary to expectation, no positive correlation between IES-R total score and the subscales was found. The correlation between VAS of global strain as perceived by staff-members and the total scale was low and only significant by trend (r = .18, p = .09).

**Table 5:** Correlations between the scales and other instruments (N = 100).

					Impact of Event Scale (IES-R)			
	VAS scale Global burden	PTSD screening	Patient satisfaction		Intrusion	Avoidance	Hyper-arousal	IES-R total
Humiliation	0.83***	0.58***	-0.35***		0.05	0.02	-0.1	-0.08

Phys. adv. Effects	0.35***	0.31**	-0.32**		0.04	0.09	0.06	0.08

Separation	0.54***	0.47***	-0.18		-0.08	0	-0.09	-0.07

Neg. environment	0.36***	0.40***	-0.28**		-0.05	0.02	0.06	0.06

Fear	0.30**	0.46***	-0.03		0.19	0.08	-0.03	-0.01

Coercion	0.58***	0.49***	-0.27**		0.05	0.01	0.05	0.04

Total scale	0.79***	0.64***	-0.38***		0.04	0.04	-0.05	-0.03

*** p < .001 ** p < .01 * p < .05

### Cut-off value for traumatisation on CES total score

Inserting the cut-off point on the PTSD screening (4.00) led to a critical value on the "Coercion Experience Scale" of 70.

## Discussion

The results of the study yielded a six-factor solution with the factors "Humiliation" "Physical adverse effects", "Separation", "Negative environment", "Fear", and "Coercion". These factors explained 54.5% of the total variance of intercorrelations.

Cronbach alpha ranged from 0.67 to 0.93, which can be interpreted as a high internal consistency of the single factors. The highest internal consistency reached "Humiliation" (0.93), followed by "Separation" (0.92), "Coercion" (0.83), "Negative environment" (0.78), "Physical adverse effects" (0.72) and least "Fear" (0.67).

Except for the high intercorrelation (0.84) between "Separation" and "Coercion" the subscales show for the most part low to moderate intercorrelations (0.22 - 0.64), indicating an adequate independence of the respective subscales

To determine the convergent validity of the "Coercion Experience Scale" the correlations between this questionnaire and a visual analogue scale assessing the global strain during the same index-intervention was used. The analysis of correlation yielded a highly significant result (r = .79, p < .001). However, there was no significant correlation between the "Coercion Experience Scale" and the Impact of Event-Scale. Probably, this fact can be attributed to adaptation to the traumatic impact of coercive interventions, because the IES-R interview was the only scale assessed one year after the index-intervention and only 3 patients could be diagnosed with PTSD after assessment with the IES-R. Discriminant validity was measured by patient satisfaction and correlated negatively with the "Coercion Experience Scale" (r = -.38, p < .001).

The correlation between the "Coercion Experience Scale" and a screening instrument for PTSD was high (r = .64, p < .001). Together with the result mentioned above this supports the conclusion of convergent validity. Furthermore, the defined cut-off value of the screening on PTSD was used to estimate a critical point of strain induced by the coercive intervention. The regression showed that a global score of more than 70 seemed to indicate a highly restrictive measure. This cut-off point has to be considered as preliminary and is only an estimate of traumatisation. Due to a very low prevalence of PTSD in the follow-up (n = 3) we had to waive analyses of predictive values.

The index-intervention was additionally observed by experienced staff-members. Their assessment of the assumed global strain experienced by the patient during the index-intervention (VAS) was the external point of reference. Concurrent validity was low and only by trend significant (r = .18, p = .09). The reasons for the lower assessment by staff-members may be the difficulties in perceiving the full extent of the very subjective suffering induced by coercive interventions in general. Staff-members seem to differentiate the extend of coercion related to the multitude of coercive interventions carried out by them. They may set a maximum of restrictiveness at a seldom occurring intervention during which they had to forcefully overpower a severely agitated, highly aggressive patient with the help of policemen not being able to prevent the patient from injuries while on the other hand patients may feel already heavily traumatised by the circumstance of being led to the seclusion room and being locked in.

The present study has several limitations. Firstly, the study was conducted in a hospital located in a rural area with a high socioeconomic standard in South Germany, which is not representative compared to more populated areas. In other facilities with a different practice applying coercive interventions strains and restrictive experiences might be somewhat different.

Secondly, it is possible that patients might have overreported the intensity of their experiences on both the restrictions and the VAS in order to emphasise the necessity to reduce coercive interventions.

Thirdly, the sample size with 102 analysed patients is rather small. The proportion between number of patients and items is inappropriate. However, according to the Kaiser-Meyer-Olkin criterion the data were suitable for this PAF. Furthermore, the six factor solution accounted for 54.5% of the total variance of intercorrelations which is a good result in consideration of the sample size.

Fourthly, it may be contradictive to validate this questionnaire with strict statistical methods and to rely on subjective instruments like patient satisfaction and IES-R. For example, patient satisfaction and perceived coercion may be not two theoretically completely unrelated constructs as demanded for discriminant validity. Discriminant validity may therefore be questionable. On the other hand, there is no better instrument for discriminant validity than patient satisfaction which is validated itself. This objection leads to the problem that there is no gold standard on this regard.

Fifthly, the same problem occurred concerning the reliability. As there is no gold standard in this respect to come into consideration the alternate forms method for measuring reliability was excluded. However, the single factors showed a high internal consistency, which may be an estimate for reliability.

From an ethical point of view the subject of this questionnaire is more than overdue to be examined more exactly, at the same time research in this field has to deal with subjective assessment of scientifically not exactly definable variables such as human rights. The appraisal of this questionnaire has to consider the relation to the psychiatric surrounding and the ethical complexity. Although further research is urgently needed, we assume that the CES is an important scale to fill in the gap between scientific research and ethical founded constructs in psychiatry.

## Conclusions

This questionnaire is the first instrument to measure the psychological impact during psychiatric coercive interventions. Due to the ethical complexity, the psychiatric emergency situation, and the fact that no other validated instruments exist on this issue reliability and validity are subjects to restrictions, but can be estimated as satisfying. The data showed a six-factor solution. For purposes of research it can be used to compare different coercive interventions. In clinical practice it can be used as a screening instrument for patients who need support after coercive interventions to prevent consequences from traumatic experiences. The instrument has been designed for the comparison of seclusion and mechanical restraint. This should be taken into account if other interventions such as physical restraint (holding a patient on the floor without mechanical devices) or compulsory medication are assessed. Further research is needed to identify possible diagnostic, therapeutic or prognostic implications of the total score and the different subscales.

## Competing interests

The authors declare that they have no competing interests.

## Authors' contributions

JB conception and design, acquisition of data, interpretation of data, drafting the manuscript. EF analysis and interpretation of data, drafting the manuscript. TS conception and design, interpretation of data, revising the manuscript critically for important intellectual content.

## Pre-publication history

The pre-publication history for this paper can be accessed here:

http://www.biomedcentral.com/1471-244X/10/5/prepub
